# Echocardiographic Evaluation of Indices of Severity of Pulmonary Stenosis in Dogs: Reproducibility and Effects of General Anesthesia

**DOI:** 10.1111/jvim.70003

**Published:** 2025-02-26

**Authors:** Evan S. Ross, Lance C. Visser, Lalida Tantisuwat, Khursheed Mama, Brianna M. Potter, Brian A. Scansen

**Affiliations:** ^1^ Department of Clinical Sciences, College of Veterinary Medicine and Biomedical Sciences Colorado State University Fort Collins Colorado USA

**Keywords:** canine, measurement variability, orifice area, pulmonic stenosis, repeatability

## Abstract

**Background:**

The effects of general anesthesia (GA) on less flow‐dependent (velocity ratio, velocity time integral [VTI] ratio and indexed pulmonary valve area [iPVA]) and flow‐dependent (mean [PVmeanPG] and maximum pressure gradient [PVmaxPG]) indices of severity of pulmonary stenosis (PS) are unclear.

**Objectives:**

Determine the effects of GA on indices of severity of PS in dogs undergoing an interventional procedure (IP). Determine the reproducibility of indices of severity of PS.

**Animals:**

Thirty‐nine dogs with PS.

**Methods:**

Prospective cross‐sectional study. Five repeated echocardiograms were performed over 3 days. Day 1: two echocardiograms were performed by 2 different operators. Day 2: echocardiograms were performed before and after GA but before IP. Day 3: an echocardiogram was performed after the IP.

**Results:**

After GA, median (IQR) cardiac index (2.1 [1.6–2.6] L/min/m^2^), PVmeanPG (45.0 [26.0–55.2] mmHg), PVmaxPG (76.6 [46.6–100.3] mmHg) were decreased (*p* ≤0.001) compared to before GA (2.8 [2.2–3.0] L/min/m^2^, 55.9 [47.6–73.1] mmHg, 96.1 [81.6–127.0] mmHg, respectively). There were no differences (*p* ≥0.35) in velocity ratio, VTI ratio, or iPVA after GA. Intra‐operator and inter‐operator coefficients of variation (95% CI) were highest for iPVA (13.8% [10.4–18.4] and 13.5% [11.0–18.4], respectively) and lowest for velocity ratio (9.2% [7.7–12.3] and 9.3% [7.7–12.4], respectively).

**Conclusions and Clinical Importance:**

PVmeanPG and PVmaxPG might be misleading in states of reduced flow. An integrative assessment of severity of PS that includes less flow‐dependent indices is recommended. Reproducibility of indices of severity of PS should be considered when re‐evaluating dogs with PS.

**Abbreviations**
AV: aortic valve
CSA: cross‐sectional area
CV: coefficient of variation
GA: general anesthesia
i2D_TAPSE: two‐dimensional tricuspid annular plane systolic excursion indexed to body weight
iFAC: right ventricular fractional area change indexed to body weight
IP: interventional procedure
iPVA: pulmonary valve area indexed to body surface area
iRAA: right atrial area indexed to body weight
iRVAd: right ventricular area at end‐diastole indexed to body weight
iRV_S': right ventricular systolic myocardial velocity at the lateral tricuspid annulus indexed to body weight
iRVWT: right ventricular wall thickness indexed to body weight
PFO: patent foramen ovale
PS: pulmonary stenosis
PV: pulmonary valve
PVmaxPG: maximum transpulmonary pressure gradient
PVmeanPG: mean transpulmonary pressure gradient
RC: reproducibility coefficient
RV: right ventricle/ventricular
Vmax: maximum velocity
VTI: velocity time integral

## 1 Introduction

In dogs, the severity of pulmonary stenosis (PS) and decision to perform or repeat a transcatheter interventional procedure (IP) are strongly dependent on the Doppler echocardiography‐derived maximum transpulmonary pressure gradient (PVmaxPG) and the presence of clinical signs. However, many dogs with PS do not show clinical signs and pressure gradients across stenotic valves can be misleading. Pressure gradients are influenced by transpulmonary blood flow (i.e., they are flow‐dependent) and the degree of stenosis (i.e., valve orifice area) [1, 2, 3, 4, 5]. Increased sympathetic tone and positive inotropic drugs might increase PVmaxPG and overestimate severity of PS. Right ventricular (RV) systolic dysfunction, drugs that block the beta‐adrenergic receptor, and the myocardial‐depressant effects of general anesthesia (GA) might decrease PVmaxPG and underestimate of severity of PS. The American Society of Echocardiography's recommendations for the echocardiographic assessment of aortic valve (AV) stenosis discourage relying solely on transvalvular velocities or pressure gradients [6]. An integrative approach that includes routine measurement of at least one less flow‐dependent method, for example, estimated valve area, based on the continuity equation, is recommended.

Less flow‐dependent indices of severity of PS have been evaluated in dogs with PS. These indices are derived from the continuity equation and include Doppler‐derived estimates of pulmonary valve area indexed to body surface area (iPVA), velocity time integral (VTI) ratios, and velocity ratios. The velocity and VTI ratios, also referred to as dimensionless indices, compare spectral Doppler profiles of the AV and pulmonary valve (PV). They reduce the potential for measurement error by eliminating AV cross‐sectional area (CSA_AV_), which is estimated from its squared diameter (radius), using the continuity equation. Following atenolol and reduction in RV systolic function and cardiac index, iPVA, VTI ratio and velocity ratio did not change but the flow‐dependent indices, mean and PVmaxPG, were reduced [5]. Use of less flow‐dependent indices as part of an integrative assessment of severity of PS is being increasingly reported in dogs [7, 8, 9, 10, 11, 12].

The cardio‐depressant effects of GA and the measurement variability of indices of severity of PS have not been systematically evaluated in dogs with PS. Further understanding of the degree of flow‐dependence (or lack thereof) and the effects of GA on the various indices might aid interpretation of severity of PS during states of altered blood flow and GA. Evaluating the intra‐operator and inter‐operator reproducibility of indices of severity of PS might help guide clinical decision‐making regarding assessment of progression or regression of disease during serial examinations, and especially the conundrum of re‐stenosis post‐intervention.

We sought to determine the effects of GA on indices of severity of PS in dogs with PS undergoing an IP, that is, balloon pulmonary valvuloplasty or transpulmonary stent implantation. We also sought to determine the reproducibility (intra‐operator and inter‐operator variability) of these indices in dogs with PS. We hypothesized that GA would have a greater effect on flow‐dependent indices of severity of PS (mean transpulmonary pressure gradient [PVmeanPG], PVmaxPG) than less‐flow dependent indices (Velocity ratio, VTI ratio, and iPVA) in dogs with PS.

## 2 Materials and Methods

### 2.1 Animals

Study procedures were approved by the Institutional Animal Care and Use Committee and Veterinary Teaching Hospital Clinical Trial Review Board at Colorado State University (protocol #3355). All dog owners provided written consent prior to enrolling their dogs in the study.

Study subjects consisted of client owned dogs that were presented to our hospital for evaluation of suspected cardiac disease or had PS diagnosed elsewhere and were referred for an IP (balloon pulmonary valvuloplasty or transpulmonary stent implantation). Following a review of their clinical history, physical examination, and echocardiographic examination performed for clinical purposes, dogs were consecutively enrolled over a 12‐month period if they were diagnosed with PS and had no additional hemodynamically relevant cardiac abnormalities other than those related to PS, had a PVmaxPG >50 mmHg, and were scheduled to undergo an IP. Dogs were excluded if they had a sustained clinically relevant arrhythmia (e.g., atrial fibrillation), and had evidence of systemic disease (based on the history and physical examination), not considered to be secondary to PS. Dogs administered a cardiac medication (e.g., atenolol) were not excluded. Medication dosages were kept unchanged throughout the study period unless the attending clinician(s) felt a change in dose was necessary for optimal patient care. Dogs diagnosed with a patent foramen ovale (PFO) were not excluded provided their shunting was considered mild (based on color Doppler imaging) and PCV was <60%. Dogs diagnosed with a coronary artery anomaly were not excluded. Dogs with tricuspid regurgitation were not excluded provided the cause of the tricuspid regurgitation was considered secondary to PS and not overt tricuspid valve dysplasia. Dogs were considered to have tricuspid valve dysplasia and excluded if there were obvious abnormalities to the tricuspid valve apparatus including overt valvular thickening and irregularities, abnormal valve motion (e.g., septal leaflet tethering or stenosis), or overt chordal abnormalities such as shortened/blunted chordae +/− thickening.

### 2.2 Study Design

**FIGURE 1:**
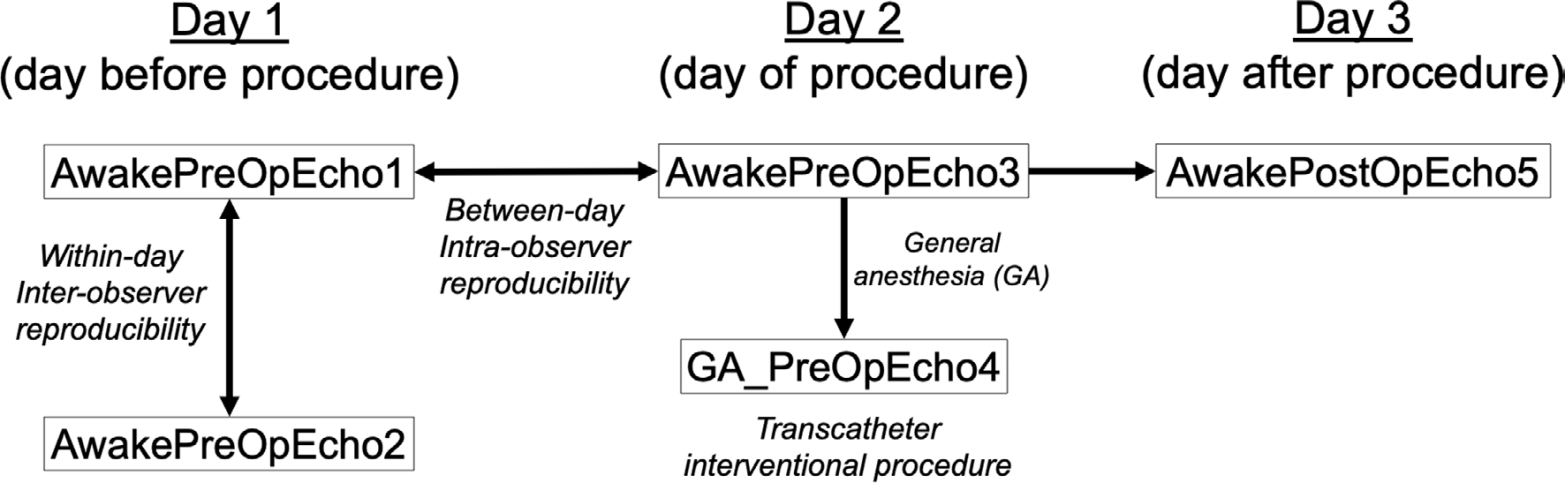
A flow chart indicating sequence of events for dogs enrolled in the study. All dogs underwent six echocardiographic examinations. On day one (the day before the transcatheter interventional procedure), dogs underwent two consecutive echocardiographic examinations. One was performed by a cardiology resident (AwakePreOpEcho1) and the other by a cardiologist (AwakePreOpEcho2) to assess inter‐operator (within‐day) reproducibility. The order was determined by coin flip. The next day (day of the interventional procedure) a third echocardiographic examination was performed (AwakePreOpEcho3) to assess intra‐operator between‐day reproducibility. Dogs then underwent general anesthesia (GA) for their transcatheter interventional procedure and an echocardiographic examination was repeated (GA_PreOpEcho4) immediately prior to the transcatheter procedure. The final echocardiographic examination was performed the day after the transcatheter procedure (AwakePostOpEcho5). With the exception of AwakePreOp2, all echocardiographic examinations were acquired and measured by the same investigator, a cardiology resident. Solid black arrows represent where statistical comparisons were made.

Dogs underwent five echocardiographic examinations for study purposes (Figure 1). On day one, dogs underwent two consecutive echocardiographic examinations to assess inter‐operator (within‐day) reproducibility where one was performed by a cardiology resident (ESR; AwakePreOpEcho1) and the other by a cardiologist (LCV; AwakePreOpEcho2). The order was determined by coin flip. The next day (day of the IP) a third echocardiographic examination was performed (AwakePreOpEcho3). This echocardiographic examination was used to assess intra‐operator between‐day reproducibility and served as a baseline comparison for the post‐GA comparison. Dogs then underwent GA for their IP and an echocardiographic examination was repeated immediately prior to the IP (GA_PreOpEcho4). The final echocardiographic examination was performed after full recovery from anesthesia the day after the transcatheter procedure (AwakePostOpEcho5). With the exception of AwakePreOp2, all echocardiographic examinations were acquired and measured by the same investigator (ESR). Use of butorphanol (0.25 mg/kg IV) approximately 5‐min prior to echocardiography was permitted to help facilitate the examination provided the sedative was utilized in the same manner prior to all non‐anesthetized echocardiograms. Butorphanol was not repeated for AwakePreOp2 as it occurred immediately after AwakePreOp1.

### 2.3 Echocardiographic Examination

Echocardiographic examinations were performed using the same ultrasound unit (Philips EPIQ 7C, Philips Healthcare, Andover, MA, USA) equipped with several cardiac transducers and simultaneous ECG. Choice of carrier frequency and techniques to optimize two‐dimensional image quality were at the discretion of the operator. Data were captured digitally for measurements at an off‐cart workstation with analysis software (TomTec Imaging Systems GmbH, Chicago, IL). The same imaging protocol was used for each echocardiographic examination by each operator. Care was taken to optimize spectral Doppler recordings. The cursor was aligned as parallel as possible to blood or myocardial motion. Sweep speed (≥ 100 mm/s), baseline, scale, and gain were all manually adjusted to optimize the velocity spectra. Over‐gained signals contributing to a blooming artifact were avoided. All echocardiographic measurements were performed by the individual who acquired the images. At the time of measurement, each individual was masked to previous measurements and measurements performed by the other individual. All measurements were performed in triplicate, typically from consecutive cycles, and averaged. Specifically, three representative cycles consistency exhibiting the high quality were selected for measurement. For spectral Doppler measurements, the three fastest velocities were not necessarily sought out and measured. If a pronounced sinus arrhythmia was encountered, five cardiac cycles were averaged.

A right parasternal long‐axis left ventricular outflow view was used to measure the AV diameter. Measurements were performed shortly after valve opening and between the hinge points of the maximally opened cusps. A right parasternal short‐axis basilar view optimized for the RV outflow tract with color Doppler guidance was used to collect continuous wave Doppler systolic velocity spectra through the RV outflow tract and PV. The outer edge of the modal velocities (denser signals) from fixed obstructions was traced [6]. Signals from dynamic obstructions were not measured. Care was taken to avoid including fine linear signals (“beard”) in the measurements [13]. From the tracings the software determined maximum velocity (Vmax_PV_), PVmaxPG, PVmeanPG, and VTI_PV_. If alignment to flow was considered suboptimal, alternate imaging planes were utilized for measuring velocity spectra of the PV, for example, left cranial views. A subcostal view was used to acquire pulsed‐wave Doppler velocity spectra of the AV. The sample volume was positioned at the hinge points of the AV leaflets. Maximum velocity (Vmax_AV_) and VTI_AV_ were determined using the same technique described above. A left apical 4‐chamber view optimized for the right heart was used for measurements of right heart size and function indexed to body weight as previously described [7]. Measurements included maximum right atrial area (iRAA) using planimetry, right ventricular area at end‐diastole (iRVAd) and end‐systole using planimetry, right ventricular free wall thickness at end‐diastole at the mid‐ventricular level (iRVWT), tricuspid annular plane systolic excursion using two‐dimensional echocardiography (i2D_TAPSE) [14], and pulsed‐wave tissue Doppler‐derived peak systolic RV myocardial velocity at the lateral tricuspid annulus (iRV_S') [15, 16].

Velocity time integral ratio (VTI_AV_/VTI_PV_) was calculated as VTI_AV_ (cm) ÷ VTI_PV_ (cm). Velocity ratio (Vmax_AV_/Vmax_PV_) was calculated as Vmax_AV_ ÷ Vmax_PV_. Cross‐sectional area of the AV was calculated as: 0.785 × AV diameter (cm) [2]. The continuity equation was used to determine the effective orifice area of the PV or estimated pulmonary valve area (PVA) as follows: PVA (cm^2^) = (CSA_AV_ × VTI_AV_) ÷ VTI_PV_. The PVA was indexed to body surface area as follows: iPVA = PVA ÷ body surface area (m^2^). Body surface area was derived from body weight: 0.101 × body weight [kg]^2/3^. Stroke volume was estimated as CSA_AV_ × VTI_AV_, cardiac output (L/min) as heart rate (gathered from when the VTI_AV_ was recorded) × stroke volume, and cardiac index (CI; L/min/m^2^) as cardiac output ÷ body surface area. Indices of right heart size were indexed to body weight (kg) using previously published scaling exponents: iRAA = cm^2^/kg^0.71^, iRVAd = cm^2^/kg^0.62^, and iRVWT = cm/kg^0.25^ [15]. Percent RV fractional area change was calculated as ((RVAd—RV area at end‐systole) ÷ RVAd) × 100. Indices of RV function were indexed to body weight using previously published scaling exponents: i2D_TAPSE = mm/kg^0.284^, iFAC = %/kg^−0.097^, and iRV_S' = cm/s/kg^0.233^ [14, 16].

### 2.4 General Anesthesia

The GA protocol was designed by an anesthesiologist (KM). The GA of all patients was overseen and monitored by our hospital's Anesthesiology Service. The GA protocol was as follows: approximately 1 h following butorphanol administration provided for AwakePreOpEcho3, dogs received midazolam 0.2 mg/kg IV, fentanyl up to 15 μg/kg IV, and etomidate up to 1 mg/kg IV (diluted 1 to 4 with saline or LRS) to facilitate intubation. Atropine 0.02 mg/kg IM or titrated IV was administered at the discretion of the anesthesiologist on duty. Maropitant 1 mg/kg SC was administered following induction. Metoclopramide 0.25 mg/kg SC was added at the discretion of the anesthesiologist.

Dogs were maintained on a fentanyl constant rate infusion at 10 μg/kg/h and lidocaine infusion at 50 μg/kg/min with isoflurane (End Tidal 0.8–1.2%) during the initial period of GA and through GA_PreOpEcho4. An arterial blood gas was evaluated prior to the IP to verify that PaCO_2_ was between 35 and 50 mmHg and assess oxygenation. At the onset of the procedure, the lidocaine infusion was increased to 100 μg/kg/min with lidocaine boluses administered as necessary for arrhythmia management. Isoflurane also was adjusted as needed to maintain an appropriate plane of GA. Dopamine 5–7 μg/kg/min was administered as needed for blood pressure support. If a dog had a known PFO, phenylephrine IV was titrated as needed to improve oxygenation. On recovery, dogs received naloxone at increments of 2–5 μg/kg IV or 5–10 μg/kg SC at the discretion of the anesthesiologist. Flumazenil was also administered in case of dysphoria and dogs were tranquilized with 0.005–0.01 mg/kg of acepromazine IV if necessary. Dexamethasone 0.1–0.25 mg/kg IV was administered prior to recovery if indicated for airway management.

### 2.5 Statistical Analysis

Statistical tests were performed using commercial software (MedCalc Statistical Software, Ostend, Belgium and GraphPad Prism 10, La Jolla, CA). Data are reported as median (25th‐percentile, 75th‐percentile) unless otherwise stated. Normality testing was performed using the Shapiro–Wilk test. Comparisons of paired samples were performed with a paired t‐test or Wilcoxon test if differences were not normally distributed. Comparisons among three groups were made with a Kruskal–Wallis test and, if significant, post hoc comparisons were made using Conover test. Reproducibility of repeated measurements/calculations (intra‐operator and inter‐operator) was quantitated using coefficients of variation (CV) and the 95% reproducibility coefficient (RC). The CVs were calculated using the within‐subject standard deviation (wSD) method: (wSD ÷ overall mean) × 100. The 95% RCs were calculated as 1.96 × √ 2 × wSD [17]. Statistical significance was set at *p* < 0.05.

## 3 Results

Forty dogs were evaluated for this study. One dog was excluded because it was diagnosed with concurrent tricuspid valve dysplasia. Thus, 39 dogs participated in the study. Breeds represented were 17 French Bulldogs, 8 mixed breeds, 4 Pitbull Terriers, 2 Huskies, and one each of the following: Australian Cattle Dog, Tibetan Terrier, Golden Retriever, Jack Russell Terrier, Cavalier King Charles Spaniel, Airedale Terrier, Maltese, and an American Eskimo. Median body weight and age were 9.9 (7.2, 16.8) kg and 0.8 (0.5, 1.6) years, respectively. Nineteen were female and 20 were male. Seven dogs (18%) had a PFO. Median PCV was 43 (38, 46) %. One dog had a PCV of 61% but did not have evidence of a shunt/PFO echocardiographically or angiographically. No dogs were diagnosed with a coronary artery anomaly. Thirteen dogs (33%) were experiencing clinical signs; 7 had exercise intolerance, 6 had experienced syncope, 4 had gastrointestinal signs (considered to be secondary to their PS), and 3 had right‐sided congestive heart failure. Thirty‐five dogs (95%) received butorphanol to facilitate their echocardiographic examinations. Thirty‐eight dogs (97%) were receiving atenolol (1–2 mg/kg PO q12h) prior to study enrollment. Atenolol dose was changed by the attending clinician prior to AwakePostOpEcho5 in 9 dogs and after AwakePreOpEcho2 in 3 dogs. The latter 3 dogs were excluded from statistical analyses of intra‐operator reproducibility (AwakePreOpEcho1 vs. AwakePreOpEcho3). Other medications dogs were previously prescribed and where dosages remained consistent throughout the study were furosemide (*n* = 3), mexiletine (*n* = 1), enalapril (*n* = 1), and sildenafil (*n* = 1). Two dogs did not undergo GA_PreOpEcho4 and AwakePostOpEcho5; one because their owner decided against the IP and the other experienced severe hypoxemia under GA and the procedure was aborted. Unfortunately, in 2 dogs, AwakePreOpEcho3 failed to transfer to the imaging server and the studies were lost. Of the 35 dogs that underwent a transcatheter procedure, 23 dogs had a balloon pulmonary valvuloplasty and 12 dogs had a transpulmonary stent implantation. Four dogs did not participate in the inter‐operator reproducibility portion of the study due to logistical challenges. Four did not undergo AwakePreOpEcho2 and 2 did not undergo AwakePreOpEcho1.

Coefficients of variation and 95% RCs for intra‐operator between‐day (AwakePreOpEcho1 vs. AwakePreOpEcho3) and inter‐operator within‐day (AwakePreOpEcho1 vs. AwakePreOpEcho2) reproducibility of the indices of severity of PS and right heart size and function are shown in Table 1. For intra‐operator reproducibility, all CVs were ≤22.2%. For inter‐operator reproducibility, all CVs were ≤25.2%. With the exception of iFAC, CVs and 95% RCs were similar when comparing intra‐operator and inter‐operator reproducibility. When comparing the indices of severity of PS, the intra‐operator and inter‐operator CVs for iPVA were the highest. The minimum–maximum values for each echocardiographic variable collected from the dogs used for the reproducibility analyses were as follows: PVmeanPG 28.8–115.2 mmHg, PVmaxPG 46.6–206.3 mmHg, Vmax_AV_/Vmax_PV_ 0.14–0.40, VTI_AV_/VTI_PV_ 0.06–0.25, iPVA 0.09–0.59 cm^2^/m^2^, i2D_TAPSE 1.67–5.77 mm/kg^0.284^, iFAC 23.5–89.3%/kg^−0.097^, iRAA 0.60–2.66 cm^2^/kg^0.71^, iRVAd 0.60–3.25 cm^2^/kg^0.62^, iRVWT 0.27–1.15 cm/kg^0.25^.

**TABLE 1:** Reproducibility of echocardiographic indices of severity of pulmonary stenosis and right heart size and function from dogs with pulmonary stenosis that were examined on two different days by the same operator (intra‐operator variability) and by two different operators on the same day.

Echocardiographic variable	Intra‐operator (between‐day) reproducibility		Inter‐operator (within‐day) reproducibility	
	*n* = 32 dogs^b^		*n* = 35 dogs	
	CV (95% CI) %	95% RC (95% CI)^a^	CV (95% CI) %	95% RC (95% CI)^a^
PVmeanPG (mmHg)	10.6 (8.5, 14.2)	19.3 (15.4, 26.0)	9.6 (7.8, 12.5)	17.8 (14.5, 23.3)
PVmaxPG (mmHg)	11.5 (9.2, 15.5)	36.2 (28.8, 48.7)	9.4 (7.7, 12.3)	30.3 (24.6, 39.6)
Vmax_AV_/Vmax_PV_	9.2 (7.7, 12.3)	0.06 (0.05, 0.08)	9.3 (7.7, 12.4)	0.06 (0.05, 0.08)
VTI_AV_/VTI_PV_	12.2 (9.8, 17.1)	0.05 (0.04, 0.07)	12.4 (9.9, 17.4)	0.05 (0.04, 0.07)
iPVA (cm^2^/m^2^)	13.8 (10.4, 18.4)	0.12 (0.09, 0.16)	13.5 (11.0, 18.4)	0.11 (0.09, 0.15)
i2D_TAPSE (mm/kg^0.284^)	20.2 (15.9, 28.0)	2.2 (1.7, 3.0)	21.9 (17.7, 29.2)	2.1 (1.7, 2.8)
iFAC (%/kg^−0.097^)	16.7 (13.3, 22.6)	29.2 (23.2, 39.5)	25.2 (20.3, 33.1)	38.1 (32.6, 50.2)
iRAA (cm^2^/kg^0.71^)	12.2 (9.9, 16.4)	0.41 (0.33, 0.55)	13.0 (10.5, 17.2)	0.45 (0.36, 0.59)
iRVAd (cm^2^/kg^0.62^)	22.2 (17.5, 30.6)	0.80 (0.63, 1.10)	19.4 (15.7, 25.4)	0.68 (0.55, 0.89)
iRVWT (cm/kg^0.25^)	15.4 (12.4, 21.3)	0.26 (0.21, 0.36)	16.8 (13.7, 22.3)	0.28 (0.23, 0.37)

Abbreviations: AV, aortic valve; CI, confidence interval; CV, coefficient of variation; i2D_TAPSE, two‐dimensional tricuspid annular plane systolic excursion indexed to body weight; iFAC, right ventricular fractional area change indexed to body weight; iPVA, pulmonary valve area indexed to body weight; iRAA, right atrial area indexed to body weight; iRVAd, right ventricular area at end‐diastole indexed to body weight; iRVWT, right ventricular wall thickness indexed to body weight; PV, pulmonary valve; PVmaxPG, pulmonary valve maximum pressure gradient; PVmeanPG, pulmonary valve mean pressure gradient; RC, reproducibility coefficient; Vmax, maximum velocity; VTI, velocity time integral.

^a^ 95% RC are the same unit as the echocardiographic variable.

^b^ Three dogs received a dose change in atenolol between echocardiographic examinations and were excluded from intra‐operator reproducibility analyses.

**FIGURE 2:**
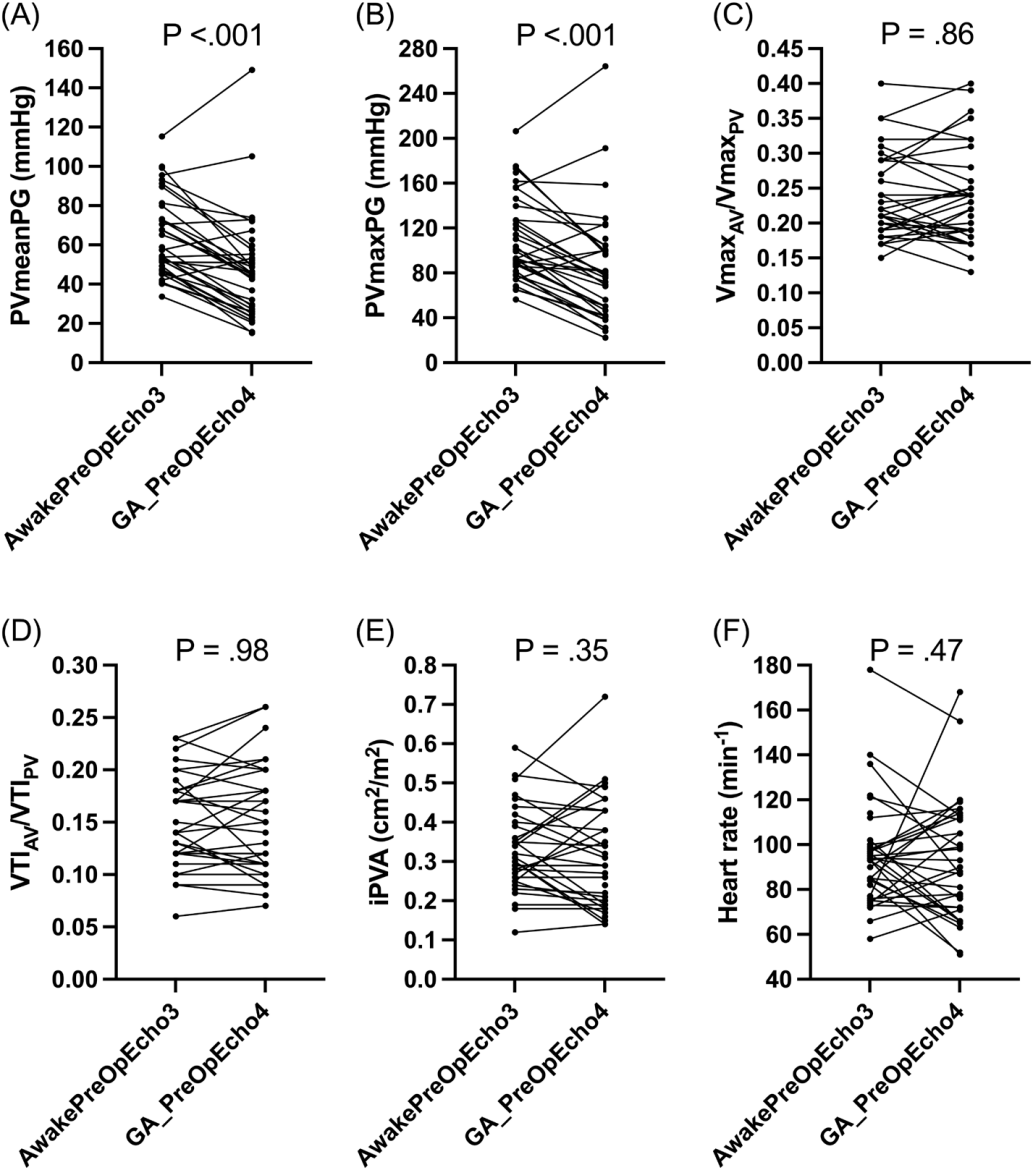
Pulmonary valve mean pressure gradient (PVmeanPG; A), pulmonary valve maximum pressure gradient (PVmaxPG; B), maximum velocity ratio of the aortic valve indexed to pulmonary valve (Vmax_AV_/Vmax_PV_; C), velocity time integral ratio of the aortic valve indexed to pulmonary valve (VTI_AV_/VTI_PV_; D), pulmonary valve area indexed to body weight (iPVA; E), and heart rate (F) before (AwakePreOpEcho3) and after general anesthesia (GA) but before a transcatheter interventional procedure (GA_PreOpEcho4).

Table 2 shows a summary of the echocardiographic data before (AwakePreOpEcho3) and after GA but prior to an IP (GA_PreOpEcho4). Cardiac index, indices of longitudinal RV systolic function (i2D_TAPSE and iRV_S'), and indices of right heart chamber size (iRAA and iRVAd) were significantly reduced (all *p* ≤0.001) after GA. Flow‐dependent indices of severity of PS (PVmeanPG and PVmaxPG) were significantly decreased (both *p* < 0.001) after GA (Figure 2). All of the less flow‐dependent indices (Vmax_AV_/Vmax_PV_, VTI_AV_/VTI_PV_, and iPVA) and heart rate were not significantly different (all *p* ≥0.35) after GA (Figure 2).

**TABLE 2:** Echocardiographic data from 35 dogs with pulmonary stenosis before (AwakePreOpEcho3) and after general anesthesia but prior to an interventional procedure (GA_PreOpEcho4).

Echocardiographic variables	AwakePreOpEcho3	GA_PreOpEcho4	*p*
Heart rate (min^−1^)	93 (77, 99)	89 (72, 112)	0.47
Cardiac index (L/min/m^2^)	2.8 (2.2, 3.0)	2.1 (1.6, 2.6)	**0.0012**
PVmeanPG (mmHg)	55.9 (47.6, 73.1)	45.0 (26.0, 55.2)	**<0.0001**
PVmaxPG (mmHg)	96.1 (81.6, 127.0)	76.6 (46.6, 100.3)	**0.0002**
Vmax_AV_/Vmax_PV_	0.22 (0.19, 0.29)	0.24 (0.19, 0.29)	0.86
VTI_AV_/VTI_PV_	0.14 (0.12, 0.18)	0.15 (0.11, 0.19)	0.98
iPVA (cm^2^/m^2^)	0.31 (0.25, 0.40)	0.31 (0.19, 0.43)	0.35
i2D_TAPSE (mm/kg^0.284^)	4.1 (3.4, 4.9)	2.7 (2.0, 3.4)	**<0.0001**
iRV_S' (cm/s/kg^0.233^)	4.0 (3.3, 4.5)	3.5 (2.6, 4.1)	**0.008**
iFAC (%/kg^−0.097^)	63.2 (52.7, 73.7)	65.6 (54.4, 78.1)	0.22
iRAA (cm^2^/kg^0.71^)	1.0 (0.9, 1.7)	0.8 (0.7, 1.0)	**<0.0001**
iRVAd (cm^2^/kg^0.62^)	1.2 (1.1, 1.4)	1.0 (0.8, 1.3)	**0.0001**
iRVWT (cm/kg^0.25^)	0.53 (0.44, 0.70)	0.56 (0.42, 0.72)	0.62

*Note:* Data summarized as median (25th‐percentile, 75th‐percentile). Bolded *p* values denote statistical significance.

Abbreviations: AV, aortic valve; i2D_TAPSE, two‐dimensional tricuspid annular plane systolic excursion indexed to body weight; iFAC, right ventricular fractional area change indexed to body weight; iPVA, pulmonary valve area indexed to body weight; iRAA, right atrial area indexed to body weight; iRVAd, right ventricular area at end‐diastole indexed to body weight; iRV_S', right ventricular systolic myocardial velocity at the lateral tricuspid annulus indexed to body weight; iRVWT, right ventricular wall thickness indexed to body weight; PV, pulmonary valve; PVmaxPG, pulmonary valve maximum pressure gradient; PVmeanPG, pulmonary valve mean pressure gradient; Vmax, maximum velocity; VTI, velocity time integral.

A summary of the echocardiographic data of the 35 dogs that underwent an IP are presented in Table 3. Heart rate, cardiac index, and all indices of RV systolic function were significantly increased (all *p* ≤0.01) post‐procedure. Flow‐dependent indices of severity of PS (PVmeanPG and PVmaxPG) were significantly improved (decreased) post‐procedure (both *p* < 0.001). All less flow‐dependent indices (Vmax_AV_/Vmax_PV_, VTI_AV_/VTI_PV_, and iPVA) were also significantly improved (increased) post‐procedure (all *p* < 0.001).

**TABLE 3:** Echocardiographic data from 35 dogs with pulmonary stenosis before (AwakePreOpEcho3) and after their transcatheter interventional procedure (AwakePostOpEcho5).

Echocardiographic variables	AwakePreOpEcho3	AwakePostOpEcho5	*p*
Heart rate (min^−1^)	93 (78, 100)	107 (87, 115)	**0.004**
Cardiac index (L/min/m^2^)	2.8 (2.2, 3.0)	3.0 (2.5, 3.4)	**0.01**
PVmeanPG (mmHg)	57.4 (48.0, 78.2)	19.8 (15.0, 25.8)	**<0.0001**
PVmaxPG (mmHg)	99.3 (82.6, 136.4)	38.9 (25.8, 53.2)	**<0.0001**
Vmax_AV_/Vmax_PV_	0.22 (0.19, 0.29)	0.33 (0.26, 0.40)	**<0.0001**
VTI_AV_/VTI_PV_	0.14 (0.12, 0.18)	0.28 (0.21, 0.32)	**<0.0001**
iPVA (cm^2^/m^2^)	0.31 (0.25, 0.40)	0.59 (0.43, 0.92)	**<0.0001**
i2D_TAPSE (mm/kg^0.284^)	4.1 (3.4, 4.9)	5.2 (4.5, 5.8)	**<0.0001**
iRV_S' (cm/s/kg^0.233^)	4.0 (3.3, 4.5)	4.6 (4.1, 5.7)	**<0.0001**
iFAC (%/kg^−0.097^)	62.1 (52.9, 73.0)	77.2 (66.9, 87.4)	**<0.0001**
iRAA (cm^2^/kg^0.71^)	1.1 (0.9, 1.6)	1.2 (0.9, 1.7)	0.26
iRVAd (cm^2^/kg^0.62^)	1.0 (1.1, 1.4)	1.3 (1.1, 1.6)	**0.002**
iRVWT (cm/kg^0.25^)	0.57 (0.44, 0.72)	0.59 (0.48, 0.72)	0.15

*Note:* Data summarized as median (25th‐percentile, 75th‐percentile). Bolded *p*‐values denote statistical significance.

Abbreviations: AV, aortic valve; i2D_TAPSE, two‐dimensional tricuspid annular plane systolic excursion indexed to body weight; iFAC, right ventricular fractional area change indexed to body weight; iPVA, pulmonary valve area indexed to body weight; iRAA, right atrial area indexed to body weight; iRVAd, right ventricular area at end‐diastole indexed to body weight; iRV_S', right ventricular systolic myocardial velocity at the lateral tricuspid annulus indexed to body weight; iRVWT, right ventricular wall thickness indexed to body weight; PV, pulmonary valve; PVmaxPG, pulmonary valve maximum pressure gradient; PVmeanPG, pulmonary valve mean pressure gradient; Vmax, maximum velocity; VTI, velocity time integral.

Percent change of the indices of severity of PS of between‐day controls (AwakePreOpEcho1 vs. AwakePreOpEcho3), after GA (AwakePreOpEcho3 vs. GA_PreOpEcho4), and after IP (AwakePreOpEcho3 vs. AwakePostOpEcho5) are shown in Table 4. For the flow‐dependent indices (PVmeanPG and PVmaxPG), the percent change of the between‐day controls, after GA, and after IP were each significantly different from each other (all *p* < 0.05). The percent change of less flow‐dependent indices, Vmax_AV_/Vmax_PV_ and VTI_AV_/VTI_PV_, were only significantly different after IP (all *p* < 0.05). For Vmax_AV_/Vmax_PV_ and VTI_AV_/VTI_PV_, no significant differences in percent change were identified when comparing between‐day controls and after GA (all *p* > 0.05). For iPVA, the percent change of the between‐day controls, after GA, and after IP were each significantly different from each other (all *p* < 0.05).

**TABLE 4:** Percent change of the indices of severity of PS of between‐day controls (AwakePreOpEcho1 vs. AwakePreOpEcho3), after general anesthesia (AwakePreOpEcho3 vs. GA_PreOpEcho4), and after an interventional procedure, that is, balloon pulmonary valvuloplasty or transpulmonary stent implantation (AwakePreOpEcho3 vs. AwakePostOpEcho5).

Index of severity of PS	Between‐day controls (%)	After GA (%)	After IP (%)	Overall *p*
PVmeanPG	−5.7 (−19.2, 5.9)	−35.4 (−50.4, −10.2)^a^	−69.8 (−75.0, −55.4)^a^, ^b^	**<0.001**
PVmaxPG	−2.9 (−15.9, 9.6)	−35.3 (−43.7, −7.7)^a^	−63.8 (−72.1, −50.1)^a^, ^b^	**<0.001**
Vmax_AV_/Vmax_PV_	1.2 (−4.3, 14.0)	0.0 (−10.9, 13.8)	41.2 (26.0, 61.3)^a^, ^b^	**<0.001**
VTI_AV_/VTI_PV_	4.8 (−4.6, 13.2)	0.0 (−10.3, 13.9)	94.0 (55.4, 126.9)^a^, ^b^	**<0.001**
iPVA	10.4 (1.7, 25.7)	−4.3 (−21.6, 12.5)^a^	109.2 (75.9, 163.4)^a^, ^b^	**<0.001**

*Note:* Data summarized as median (25th‐percentile, 75th‐percentile). Bolded *p*‐values denote statistical significance.

Abbreviations: AV, aortic valve; GA, general anesthesia; IP, interventional procedure; iPVA, pulmonary valve area indexed to body weight; PS, pulmonary stenosis; PV, pulmonary valve; PVmaxPG, pulmonary valve maximum pressure gradient; PVmeanPG, pulmonary valve mean pressure gradient; Vmax, maximum velocity; VTI, velocity time integral.

^a^ Significantly different from between‐day controls.

^b^ Significantly different from after GA.

The supplementary table shows the echocardiographic data of the 35 dogs that underwent an IP broken down by type, balloon valvuloplasty or transpulmonary stent implantation.

## 4 Discussion

Our results suggest that in dogs with PS, velocity ratio, VTI ratio, and iPVA are less flow‐dependent and are primarily affected by changes in valve orifice area. Pressure gradients (PVmeanPG and PVmaxPG) are dependent on transpulmonary blood flow and orifice area. Our study also helps to clarify the degree of measurement variability inherent to repeated measurements of indices of severity of PS on different days by the same operator and by different operators. Our study and others [5, 9] suggest that clinicians should utilize an integrative echocardiographic approach and incorporate at least one less flow‐dependent index when assessing severity of PS. Solely relying on pressure gradients to determine progression or regression of disease over time, after IP, or both might be misleading.

Short‐term atenolol (2–4 weeks) significantly decreases PVmeanPG and PVmaxPG but does not effect velocity ratio, VTI ratio and iPVA [5]. Our study was designed, in part, to attempt to further decrease transpulmonary flow through the potent cardio‐depressant effects of GA in order to further challenge the less flow‐dependent indices (velocity ratio, VTI ratio, iPVA) [18, 19, 20, 21, 22]. Our results support this anticipated effect of GA. Indices of longitudinal RV systolic function (i2D_TAPSE and iRV_S') were significantly decreased after GA. Also, cardiac index was decreased to a greater degree after GA in our study (mean 2.1 L/min/m^2^) compared to after atenolol in the previous study (mean 3.1 L/min/m^2^) [5]. Despite this greater reduction in transpulmonary flow, velocity ratio, VTI ratio, iPVA remained unaffected. Pressure gradients were significantly decreased (median percent change for PVmeanPG and PVmaxPG was −35%) despite no true change in severity of PS.

As proof‐of‐principal, we demonstrated that after an IP and presumably a consequent increase in valve orifice area, all indices of severity of PS significantly improved. Thus, iPVA and its simplified surrogates (velocity ratio and VTI ratio) are more likely to track changes in valve area and less likely to change in states of altered (decreased) flow when compared to pressure gradients. These results further support the notion that velocity ratio, VTI ratio, and iPVA are reliable indices of severity of PS when valve area and flow are altered, as with GA.

Several studies have highlighted the importance of RV systolic dysfunction in dogs with PS [7, 8, 12, 23]. Our results suggest that PVmeanPG and PVmax PG might be less reliable in the setting of reduced RV systolic function (in this case following GA) when compared to velocity ratio, VTI ratio, and iPVA. After GA, we observed significant decreases in 2 indices of RV longitudinal systolic function (i2D_TAPSE and iRV_S') which likely played a role in decreasing cardiac index and thus transpulmonary pressure gradients despite no change in valve area. These findings highlight an advantage inherent in velocity ratio, VTI ratio, and iPVA which, by definition, consider flow (stroke volume) and thus myocardial function when assessing severity of PS.

Our study also was designed to evaluate measurement variability and specifically the reproducibility of echocardiographic indices of severity of PS, RV systolic function, and right heart size in dogs with PS. Reproducibility can be defined as variability of the same measurement made on the same subject under changing conditions [24], that is, different days (intra‐operator, between‐day) and different operators/sonographers (inter‐operator, within‐day). Each specific measurement included repeated echocardiographic acquisitions and measurements and did not include repeated measurements from the same echocardiographic examination/acquisition. Our study design was intended to mimic clinical practice where serial echocardiographic examinations of dogs with PS are common, for example, after an IP and to monitor for re‐stenosis thereafter.

Evaluating the reproducibility of echocardiographic measurements with CVs and 95% RCs in dogs with PS has clinical relevance. The CVs reported herein are useful to compare the precision of indices with different units that assess the same thing, for example, indices of severity of PS, within the same study sample. In general, velocity ratio exhibited the least variability and, unsurprisingly, iPVA the most. However, the differences in CVs for the indices of severity of PS were small, especially when considering the overlap in 95% CIs. The 95% RCs can be useful to help judge effectiveness of an IP, to aid in the assessment of re‐stenosis [9, 10], or to help assess changes in right heart remodeling and RV function over time. The 95% RC values describe a minimal detectable change/difference that helps differentiate a true change (with 95% confidence) from a change due to measurement variability [17]. They can serve as meaningful guides to help judge progression or regression of disease, especially when compared to subjective assessment or arbitrary cutoffs such as successful balloon pulmonary valvuloplasty necessitating an immediate post‐balloon PVmaxPG decrease by ≥50%. Assuming transpulmonary flow is similar, our results suggest that a change in PVmaxPG by > or < 30–36 mmHg is highly suggestive of a true progression (potentially restenosis) or improvement in disease (i.e., after an IP), respectively. The 95% RC values are applicable to the data range they were derived from. Their utility in dogs with PVmaxPG outside a data range of 47–206 mmHg is uncertain. Similarly, our results only apply to dogs exhibiting sinus rhythm/arrhythmia.

A previous study evaluated reproducibility of echocardiographic variables in dogs with PS using CVs and 95% RCs [7]. It evaluated 8 dogs, whereas our study evaluated intra‐operator reproducibility and inter‐operator reproducibility in 32 and 35 dogs, respectively. The previous study only evaluated intra‐operator, within‐day repeatability, essentially a test–retest scenario, where repeated echocardiograms were performed under identical conditions only 2–4 h apart. Our study incorporated evaluations of intra‐operator (between‐day) and inter‐operator (within‐day) reproducibility. Our study design introduces greater potential for variability that was observed in most indices but is more applicable to clinical practice where dogs are re‐evaluated on different days and by different operators.

Each index of severity of PS has advantages and disadvantages. For example, PVmaxPG is efficient, relatively reproducible, and has documented clinical value in longitudinal studies [25, 26, 27]. However, our results suggest it might be misleading in settings of reduced transpulmonary flow. Estimates of pulmonary valve area using the continuity equation are theoretically sound and accurate but it has greater potential for measurement error, and some consider it cumbersome. Thus, in our view, VTI ratio and velocity ratio are appealing indices of severity of PS that can complement pressure gradients for routine assessment of PS. Our results suggest these ratios might provide a more optimal balance of efficiency, accuracy, and precision. Longitudinal outcome studies are needed to further evaluate the clinical value of indices of severity of PS, especially velocity ratio, VTI ratio, and iPVA. Pressure gradients should not be abandoned but complimented by less flow‐dependent indices when assessing the severity of PS.

The evaluation of individual dogs before (AwakePreOpEcho3) and after GA (GA_PreOpEcho4) shown in Figure 2 helps emphasize the importance of an integrative assessment of severity of PS. It is clear that not all echocardiographic indices behave as expected or similarly in all dogs. This is likely because at any given time point for any given index, measurement error or inaccuracies might occur in some dogs. Thus, solely relying on a single index, for example, PVmaxPG, to assess severity of PS is not ideal. An integrative approach will likely improve accuracy.

This study has limitations. We are unaware of a criterion standard that could have been used to determine and compare the accuracy of the echocardiographic indices of severity of PS in dogs with PS. Our study design serves as a clinically relevant alternative. Although we made a concerted effort to maintain similar doses of medications and similar anesthetic protocols for each patient, standardizing all medications, doses, and GA throughout the study period was not possible. If clinically indicated, dose adjustments and adjustments to anesthetic protocols by attending clinicians were permitted to optimize patient care. These adjustments could have impacted our results. However, comparisons made among echocardiographic variables within a time period should, in theory, have been impacted equally. We did not exclude all dogs with valvular regurgitation. Valvular regurgitation or shunts can compromise the accuracy of velocity ratio, VTI ratio, and iPVA due to unequal stroke volume through the outflow tracts [1]. Excluding dogs that had or developed valvular regurgitation during the study would have almost certainty eliminated nearly all dogs. Tricuspid regurgitation and pulmonary regurgitation are common in dogs with PS and each might be worsened after an IP to variable degrees. By definition, valveless transpulmonary stents induce severe or “wide open” pulmonary regurgitation, whereas a lesser degree of pulmonary regurgitation is expected in dogs that underwent balloon valvuloplasty. Thus, grouping these dogs similarly for comparisons of AwakePreOpEcho3 and AwakePostOpEcho5 could be considered problematic. However, echocardiographic variables behaved similarly when comparing statistically significant differences for dogs that had balloon valvuloplasty to dogs that had transpulmonary stent implantation (see Table S1). Only heart rate and cardiac index behaved differently (both showed significant increases) in dogs that had balloon valvuloplasty (*n* = 23) compared to dogs that had a transpulmonary stent implantation (*n* = 12) where no significant differences were identified. However, the latter finding can likely be explained by type II error (underpowered). Last, it is possible some dogs with a small PFO were enrolled, which could have impacted our results. This seems unlikely because dogs with erythrocytosis were excluded and the atrial septum was scrutinized with 2D and color Doppler imaging in all dogs.

In conclusion, our study suggests that PVmeanPG and PVmaxPG are flow‐dependent and potentially misleading in the setting of reduced transvalvular flow in dogs with PS. Velocity ratio, VTI ratio, and iPVA are less flow‐dependent and might be more reliable in settings of altered flow. An integrative assessment for severity of PS that incorporates at least one less flow‐dependent index alongside a pressure gradient is recommended for the echocardiographic assessment of dogs with PS. Clinicians should be mindful of the variability inherent to all indices of severity of PS when interpreting repeated echocardiographic examinations of dogs with PS (i.e., judging regression or progression of disease).

## Disclosure

Authors declare no off‐label use of antimicrobials.

## Ethics Statement

Approved by the Institutional Animal Care and Use Committee (IACUC) at Colorado State University (protocol #3355). Authors declare human ethics approval was not needed for this study.

## Conflicts of Interest

The authors declare no conflicts of interest.

## Supporting information


**Table S1.** Echocardiographic data before (AwakePreOpEcho3) and after (AwakePostOpEcho5) balloon pulmonary valvuloplasty or transpulmonary stent implantation.
