# Supplementary material for: Echocardiographic Evaluation of Indices of Severity of Pulmonary Stenosis in Dogs: Reproducibility and Effects of General Anesthesia
Source: J Vet Intern Med. 2025 Feb 26;39(2):e70003. doi: 10.1111/jvim.70003 (PMC11863216; doi:10.1111/jvim.70003)
Supplement: Supplementary file 1 — Table S1. Echocardiographic data before (AwakePreOpEcho3) and after (AwakePostOpEcho5) balloon pulmonary valvuloplasty or transpulmonary stent implantation. [file JVIM-39-e70003-s001.docx]

| **SUPPLEMENTARY TABLE**. Echocardiographic data before (AwakePreOpEcho3) and after (AwakePostOpEcho5) balloon pulmonary valvuloplasty or transpulmonary stent implantation. | | | | | | |
| --- | --- | --- | --- | --- | --- | --- |
| Echocardiographic variables | Balloon valvuloplasty (n = 23) | | | Transpulmonary stent (n = 12) | | |
|  | AwakePreOpEcho3 | AwakePostOpEcho5 | P-value | AwakePreOpEcho3 | AwakePostOpEcho5 | P-value |
| Heart rate (min^-1^) | 95 (80, 100) | 105 (88, 120) | .**001** | 93 (81, 100) | 111 (94, 113) | .21 |
| Cardiac index (L/min/m^2^) | 2.8 (2.4, 3.3) | 3.1 (2.8, 3.8) | **.004** | 2.2 (1.8, 2.9) | 2.5 (2.0, 2.9) | .41 |
| PVmeanPG (mmHg) | 53.3 (46.2, 77.2) | 18.6 (14.1, 23.6) | **<.001** | 67.6 (53.3, 76.3) | 25.8 (17.1, 30.9) | **<.001** |
| PVmaxPG (mmHg) | 92.2 (78.2, 131.5) | 37.1 (24.8, 47.1) | **<.001** | 105.5 (90.9, 141.5) | 46.9 (32.9, 57.7) | **<.001** |
| Vmax_AV_/Vmax_PV_ | 0.22 (0.19, 0.30) | 0.35 (0.31, 0.44) | **<.001** | 0.22 (0.20, 0.24) | 0.28 (0.24, 0.32) | **.006** |
| VTI_AV_/VTI_PV_ | 0.14 (0.12, 0.18) | 0.29 (0.25, 0.38) | **<.001** | 0.14 (0.12, 0.17) | 0.21 (0.18, 0.26) | **.001** |
| iPVA (cm^2^/m^2^) | 0.32 (0.25, 0.42) | 0.74 (0.57, 0.95) | **<.001** | 0.29 (0.25, 0.34) | 0.42 (0.38, 0.52 | **.005** |
| i2D_TAPSE (mm/kg^0.284^) | 4.2 (3.5, 4.8) | 5.2 (4.5, 6.1) | **<.001** | 3.4 (3.1, 5.0) | 5.3 (4.5, 5.8) | **.01** |
| iRV_S’ (cm/sec/kg^0.233^) | 4.0 (3.2, 4.5) | 4.6 (4.0, 5.7) | **<.001** | 4.0 (3.5, 4.4) | 4.7 (4.3, 5.6) | **.002** |
| iFAC (%/kg^-0.097^) | 57.4 (51.5, 71.5) | 76.7 (67.5, 87.5) | **<.001** | 65.0 (59.3, 74.6) | 77.2 (67.9, 86.2) | **.002** |
| iRAA (cm^2^/kg^0.71^) | 1.0 (0.9, 1.3) | 0.9 (0.9, 1.4) | .82 | 1.3 (0.9, 1.7) | 1.5 (1.2, 1.9) | .12 |
| iRVAd (cm^2^/kg^0.62^) | 1.2 (1.0, 1.4) | 1.3 (1.1, 1.6) | **.04** | 1.1 (1.0, 1.5) | 1.5 (1.2, 1.6) | **.009** |
| iRVWT (cm/kg^0.25^) | 0.49 (0.40, 0.62) | 0.55 (0.42, 0.67) | .15 | 0.69 (0.49, 0.75) | 0.67 (0.56, 0.79) | .38 |
| Data summarized as median (25^th^-percentile, 75^th^-percentile).  Abbreviations: AV, aortic valve; i2D_TAPSE, two-dimensional tricuspid annular plane systolic excursion indexed to body weight; iFAC, right ventricular fractional area change indexed to body weight; iPVA, pulmonary valve area indexed to body weight; iRAA, right atrial area indexed to body weight; iRVAd, right ventricular area at end-diastole indexed to body weight; iRV_S’, right ventricular systolic myocardial velocity at the lateral tricuspid annulus indexed to body weight; iRVWT, right ventricular wall thickness indexed to body weight; PV, pulmonary valve; PVmaxPG, pulmonary valve maximum pressure gradient; PVmeanPG, pulmonary valve mean pressure gradient; Vmax, maximum velocity; VTI, velocity time integral.  Bolded P-values denote statistical significance. | | | | | | |
